# Not on the List: A Case Report on Diabulimia and the limitations of existing classification systems

**DOI:** 10.1192/j.eurpsy.2025.1412

**Published:** 2025-08-26

**Authors:** F. M. Silva, C. Murta

**Affiliations:** 1 Department of Psychiatry and Mental Health, ULS Algarve, Faro, Portugal

## Abstract

**Introduction:**

Eating disorders have some of the highest mortality rates among mental health conditions, particularly when co-occurring with diabetes. In such cases, the risk of complications like recurrent diabetic ketoacidosis and premature mortality significantly increases. Diabulimia, characterized by intentional insulin restriction in individuals with type 1 diabetes to lose weight, remains unrecognized as a distinct diagnosis in psychiatric classification systems, limiting effective treatment options.

**Objectives:**

To examine the unique clinical challenges of managing diabulimia and address the diagnostic limitations in current psychiatric frameworks

**Methods:**

A comprehensive case report was conducted, which included a review of the patient’s clinical history, treatment responses, and psychosocial interventions. Additionally, a literature review was performed using multiple databases, including PubMed, PsycINFO, and Scopus, to evaluate current research on the co-occurrence of eating disorders and diabetes, with particular emphasis on diabulimia. This review helped contextualize the case findings within the broader clinical understanding of diabulimia and its management.

**Results:**

The patient, a 22-year-old woman, has a long history of type 1 diabetes and struggles with diabulimia. She is aware of the severe risks associated with insulin omission, but her desire for weight control leads her to intentionally restrict insulin, particularly during periods of emotional distress. She has a concurrent psychiatric history of depression and anxiety, which exacerbate her difficulties with diabetes management. The patient’s adherence to insulin therapy has been inconsistent, with cycles of brief compliance followed by relapse, particularly during times of heightened stress and body image dissatisfaction. Despite treatment with fluoxetine and ongoing cognitive-behavioral therapy (CBT), she continues to face significant challenges in managing both her diabetes and eating disorder. Recent interventions, including a structured fitness program and targeted psychotherapy, have yielded partial improvements, helping her develop healthier coping mechanisms and improve self-management. However, sustaining these gains remains difficult, and she continues to experience frequent hospitalizations due to metabolic decompensation.

**Conclusions:**

Diabulimia presents unique diagnostic and therapeutic challenges, primarily due to its lack of recognition as a distinct disorder within current psychiatric classification systems. This case highlights the complex interplay between eating disorders, mental health, and diabetes management, highlighting the need for an integrated, multidisciplinary approach to treatment. There is a critical need for increased awareness, the development of specific diagnostic criteria, and tailored therapeutic interventions to improve outcomes for individuals affected by this often-overlooked condition.

**Disclosure of Interest:**

None Declared

